# From “Customary Morality” to “Reflective Morality”: a physician's personalized competence development facilitated by narrative medicine

**DOI:** 10.3389/fmed.2026.1956818

**Published:** 2026-10-07

**Authors:** Jian Bai, Nannan Shi, Jiayi Cheng, Yunyi Li, Guorong She, Fanqiang Zheng, Xin Cheng

**Affiliations:** 1 School of Education, South China Normal University, Guangzhou, China; 2 School of Medicine, Jinan University, Guangzhou, China; 3 Faculty of Humanities and Arts, Macau University of Science and Technology, Macau, China; 4 Department of Nephrology, The First Affiliated Hospital of Jinan University, Guangzhou, China; 5 Department of Joint Surgery, The First Affiliated Hospital of Jinan University, Guangzhou, China

**Keywords:** competency-based medical education (CBME), John Dewey, narrative medicine, personalized competence, reflective morality

## Abstract

**Background:**

Contemporary medicine faces ethical complexity arising from biotechnological advances, the growth of patients' needs, and the occurrence of cross-cultural value conflicts. These challenges expose the limits of customary morality, an unreflective and habit-driven moral framework prevalent in traditional medical training. This situation demands a more dynamic, context-sensitive, and humanistic form of moral commitment from physicians, while also requiring the development of professional competence. Narrative medicine provides a potential solution to this problem, yet its theoretical foundation remains insufficiently articulated.

**Method:**

In this paper, we propose that John Dewey's moral growth model of “reflective morality” provides a coherent and generative foundation for narrative medicine and links this process to competence development within competency-based medical education (CBME).

**Results:**

The core tools of narrative medicine, close reading and reflective writing, are grounded in Dewey's moral development model, which encompasses habit, impulse, and deliberative intelligence. Reflection on habitual behavior during narrative practice works as a corrective to clinical habits, affective responses are examined and integrated through affiliation and reflection, and contextual deliberative intelligence is cultivated through reflective practice. These practices facilitate physicians' moral development, enable them to develop moral perceptiveness, decision-making capabilities, and initiative, and help them progress from passive moral conformity to active ethical agency, thereby enacting the transition from Dewey's philosophical “customary morality” to “reflective morality”. This framework further facilitates the cultivation of personalized competency, a dimension of professional competence essential to patient-centered care yet resistant to conventional training.

**Conclusion:**

This paper provides a Deweyan theoretical foundation for narrative medicine by interpreting its reflective practices through Dewey's distinction between customary and reflective morality. It proposes a habit–impulse–intelligence framework to explain the moral developmental mechanisms through which narrative medicine may influence physicians' professional formation, connecting this process to the cultivation of personalized competence.

## 1 Introduction

Since its inception, medicine has been both a technical science and a humanistic practice carrying profound moral commitments. Although technical skill transmission has been central to the medical educational system, medical training is a process of moral enculturation. The medical school functions as a moral community, transmitting normative rules regarding behavior and emotions to its trainees (1). The Hippocratic oath of “Primum non nocere” (First, do no harm) and Dr. Trudeau's maxim, “To cure sometimes, to relieve often, to comfort always,” both underscore the central role of morality and humanity in medical practice. Edmund D. Pellegrino, a pioneer in bioethics and philosophy of medicine, noted that medicine is “the most humanistic of the sciences and the most scientific of the humanities” (2). These considerations place greater demands on medical professionals training, requiring the development of physician's competence and the cultivation of moral perception, contextual sensitivity, and humanistic commitment that define truly patient-centered care, which extends beyond technical expertise.

Competence is the central aim of medical training. Epstein & Hundert defined competence as the habitual and judicious use of communication, knowledge, technical skills, clinical reasoning, emotions, values, and reflection in daily practice for the benefit of the individual and community (3). This comprehensive definition proved difficult to operate. Later, medical competence was conceptualized at three levels, canonical, contextual, and personalized (4, 5). The canonical level refers to the standards of practice that graduates must meet in a specialty. The second layer is context-dependent knowledge, skills, and attitudes, visible through practice in health care. The third layer includes personal skills, interests, habits and convictions, integrated with one's personality. Learners shape their knowledge through personal experiences, mediated by sensory, neural, and cognitive systems, in interaction with a social environment. Beyond technical skills, moral perception, humanistic literacy, reflective capacity, contextual judgment, and decision-making abilities are all among such assets, which add quality to performance. These qualities are expressed through attitudes, behaviors, and experience, and are essential to professional development, yet remain difficult to cultivate through formal training. Healthcare educators are keen to find effective ways to cultivate these competencies. With a deeper understanding of personalized competence, theoretical perspectives, such as the construct of Perezhivanie, cognitive load theory, self-determination theory, and Maslow's motivational theory, support both the shared characteristics and the diversity of personalized competency (5).

Within this broader conception of competence, personalized competence refers to the individualized manner in which professional competence is expressed, grounded in adequate knowledge and skills, while emphasizing an individual's personal style of health care provision. Personalized competence may be reflected in an individual's distinctive professional style and efforts to excel in work, as observed within the professional environment through formal and informal recognitions (5). Therefore, personalized competence is not a single and static ability, and should not be limited to several dimensions. It concerns how physicians develop and organize professional dispositions through experience and how these dispositions are adapted to the demands of clinical situations. This personalized dimension is especially relevant to patient-centered care because physicians may encounter circumstances in which standardized rules or routines do not fully determine an appropriate response. Their personalized competences are built on and expand personal strengths, contributing to personal growth and career development.

To navigate the moral complexities encountered in clinical setting effectively, physicians must develop not merely technical proficiency but also a more comprehensive capacity for moral perception, ethical judgment, and contextually appropriate action, which are core domains of professionalism (3). Unfortunately, traditional medical education and practice models typically overemphasize knowledge transmission and skill acquisition, while falling short in cultivating such capacities (6). Among emerging pedagogical approaches, narrative medicine stands out as uniquely positioned to address these moral deficits.

In 2001, Rita Charon first introduced “narrative medicine,” defining it as “the capacity to recognize, absorb, interpret, and be moved by the stories of illness,” and emphasizing that its ultimate aim is to achieve “more effective medical care” (7). Narrative medicine emphasizes cultivating the “attention–representation–affiliation” capacities of physicians through close reading and reflective writing, however, a fundamental question remains: How can these internal reflective activities translate into ethical actions in clinical practice? The moral philosophy proposed by John Dewey, the American pragmatist philosopher and educator, offers an insightful theoretical framework for addressing the practical logic of narrative medicine.

In this paper, we conceptually articulate a Deweyan moral-philosophical foundation for narrative medicine, develop a habit–impulse–intelligence model of reflective moral development, and link this process to the cultivation of personalized competence in contemporary medical education.

## 2 Practice complexity in current medicine causes ethical challenges in residents training

Modern medicine faces a variety of unprecedented complexities. Some are caused by advances in science and technology, such as gene editing and organ transplantation, which have brought about significant ethical challenges regarding the sanctity and manipulation of life; patients' medical literacy and autonomy, strengthened by the widespread use of the Internet and artificial intelligence (AI), have complicated decision-making between doctors and patients. Some conflicts arise from the interweaving of diverse cultures and religious beliefs, the clinical practical environments. These complexities create dilemmas in medical practice.

### 2.1 External ethical challenges

Ethical dilemmas in clinical practice include but are not limited to resource scarcity and fairness, confidentiality and disclosure, cultural issues and disagreement within families, end-of-life issues, and so forth. These constitute major challenges for physicians, especially for the trainees at early stages of their career. A considerable proportion of the dilemmas concerned situations with insufficient medical resources, and physicians had to compromise the quality of care under the principle of distributive justice (8). Especially in low-income countries, patients may have competing needs facing insufficient treatment resources (9), and the financial burden of treatment on economically disadvantaged patients may further complicate clinical decision-making. Other substantial dilemmas concerned confidentiality and disclosure of relevant information, patient's capacity to consent, cultural issues and disagreement within families (10), as well as end-of-life issues, and so forth.

### 2.2 Internal well-being

Another challenge faced by healthcare professionals concerns their internal well-being. Residency burnout has been a growing concern since the 1970s, exacerbated by advancements in medicine, technology, and global events such as the COVID-19 pandemic (11). Well-being is broadly conceptualized and includes multiple psychological dimensions, including residents' self-awareness and inner harmony. Medical students and junior practitioners are particularly challenged by morally distressing situations. Among 45 surveyed Canadian residents, moral distress was reported and found to be greater during intensive care unit rotations and was associated with burnout and an intention to leave the job (12). Secondary traumatic stress can lead to symptoms such as those associated with post-traumatic stress disorder. A review study highlighted the need for interventions addressing residents' burnout and promoting well-being, with varied approaches across specialties and institutions (11).

### 2.3 Challenges to the current medical education system

These challenges pose higher requirements for cultivating physicians with the capacity to harmonize scientific knowledge with ethical wisdom and humanistic commitment. They are expected to integrate bioethical principles with clinical acuity, engage in reflective practice when confronting value conflicts, and cultivate the moral courage necessary for advocating for patient interests amid institutional and resource constraints (13). The conventional medical curricula focus more on instilling principles and statutes, resulting in what Hafferty and Franks described as the transmission of a distinctive medical morality through the informal or hidden curriculum rather than conscious ethical reasoning (1, 14). Physicians frequently rely on experiential “habits” and professional “conventions” accumulated through apprenticeships. These practices characterize unreflective moral habituation. When confronted with complex ethical dilemmas, such conventional approaches may prove inadequate.

## 3 Narrative medicine as a catalyst for cultivating personalized competence

As an interdisciplinary approach integrating literature and medicine, narrative medicine is committed to patient-centered care, examining suffering, illness, disability, personhood, therapeutic relationships, and meaning in health care. Rather than treating ethics as abstract principles to be memorized, this pedagogic program engages physicians in concrete, lived experiences of patients and physicians themselves, fostering moral sensitivity and judgment through practice.

### 3.1 Core tools of narrative medicine

The core practical tools of narrative medicine are “close reading” and “reflective writing”. Rita Charon referred to close reading as the “signature method” of narrative medicine. By drawing upon literary criticism techniques, it involves in-depth analysis of patient narratives, including medical records, oral histories, illness narratives found in literature, and other forms of narrative meterial. Practitioners attend carefully to linguistic details, metaphors, narrative structures, and even silences and gaps. The purpose is to more fully apprehend and represent the patient's unique illness experience, emotional world, and value system, thereby deepening the physician's capacity for empathic understanding and contextual moral reasoning (15). Reflective writing, the second foundational tool, encourages physicians to engage in deliberate self-examination by writing about their own clinical experiences, emotional responses, ethical dilemmas, and interactions with patients and colleagues (16, 17). Through this process of structured reflection, physicians can explore the meaning embedded in their experiences, clarify their values, examine their professional roles, and develop greater self-awareness, all of which may contribute to mature moral agency in medicine.

### 3.2 Narrative medicine provides innovative methods for cultivating clinical competence

Through systematic and rigorous training in narrative skills such as close reading, reflective writing, and narrative engagement with patients, the competencies of physicians and medical students may be cultivated. A study examined the impact of narrative medicine training on the clinical skills of medical students, focusing on ACGME (the US Accreditation Council for Graduate Medical Education)/RCPSC core competencies, including communication, collaboration, and professionalism. After a one-month elective comprising close reading, reflective writing, and clinical discussions, although limited in sample size, the pilot study showed that the students reported enhanced communication skills, empathy, and perspective-taking abilities. Follow-up data after 1.5 years revealed the trainees' sustained use of narrative skills in residency, including attentive listening and incorporating emotional dimensions into patient care, suggesting sustained use of narrative approaches relevant to these core competencies (18). A Taiwanese study investigated how narrative medicine influences the professionalism of medical trainees, a core ACGME competency (19). Among 30 trainees participating in a narrative medicine program featuring reflective writing and small-group discussions, researchers identified three key mechanisms: self-exploration, reflection, and awareness of professional identity. These mechanisms were associated with reported outcomes in empathy, communication skills, doctor-patient relationships, and patient understanding. The study suggested that narrative medicine may support professionalism competence through a pathway in which narrative practice fosters self-awareness and reflection, which in turn may contribute to professional identity, ultimately supporting the essential interpersonal and ethical dimensions of clinical competence. Current forms of narrative medicine draw on a range of theoretical fields, including narratology, literary theory, continental philosophy, social constructionism, and postmodernism (20). These existing bodies of scholarship have documented the educational and empathic value of narrative medicine, yet the mechanisms through which narrative practices translate into moral agency and professional formation remain insufficiently theorized.

## 4 A Deweyan framework for narrative medicine

Narrative competence is the ability to acknowledge, absorb, interpret, and act on stories. With narrative competence, healthcare practitioners can reach and join their patients in illness, observe and articulate their personal journeys through medicine, and acknowledge kinship with and duties toward other healthcare professionals. Dewey distinguished between “customary morality” and “reflective morality,” emphasizing that morality is experiential, developmental, and situational. In his view, moral conduct arises from the dynamic interaction of “habits,” “impulses,” and “intelligence/deliberation” within an individual. Moral growth, therefore, is not a matter of adhering to fixed rules but rather an ongoing process of adjusting behavioral dispositions through reflection on experience. This process enables individuals to transcend passively accepted customary morality and construct an actively engaged reflective morality (21). This distinction provides a conceptual framework for understanding how narrative medicine facilitates physicians' progression from unreflective adherence to deliberate ethical practice.

### 4.1 Customary morality and its limitations in clinical practice

Customary morality originates from long-standing norms, traditions, and authoritative expectations upheld by specific social groups, such as professional communities. In Dewey's view, it “relies heavily on acts of praise and blame to perpetuate itself,” operates “largely through habit rather than through choice,” and enforces “unquestioning obedience to its precepts” (21). Individuals may therefore absorb and internalize these norms with limited critical examination. Medicine is strongly grounded in experience, and physicians may exhibit habitual patterns of judgment and action through their daily practices, professional conventions, and apprenticeship. Such patterns can be useful for maintaining efficiency and consistency in routine situations. However, when these habits are applied without sufficient reflection, they may resemble the unreflective features of customary morality described by Dewey. Customary morality may become insufficient when physicians are confronted with novel, complex, and value-laden clinical dilemmas. It may limit physicians' moral agency and creativity and lack the contextual sensitivity needed to navigate ethically ambiguous situations. This results in disease-centered rather than patient-centered care when individual differences, values, and circumstances are insufficiently considered. Thus, the limitation of customary morality in medicine does not lie in the existence of professional norms or habits themselves, but in their uncritical application when circumstances require reflection and adaptation. In terms of intellectual flexibility and moral responsiveness, such habitual modes of practice may therefore become constrained in complex clinical settings (Table 1). These behavioral outcomes may also contribute to professional burnout and a heightened sense of moral distress among physicians (1).

**Table 1:** Clinical patterns illustrating unreflective habitual practice.

Clinical customary norms	Manifestations	Clinical examples
Clinical inertia	An over-reliance on procedural behavioral patterns formed by clinical pathways, guidelines, etc., which may neglect patients' individual differences and unique needs, leading to “one-size-fits-all” medical treatment (22)	Some doctors habitually prescribe imaging tests for all patients with back pain, instead of assessing individual patients before deciding whether imaging tests are necessary.
Routinized communication	Using rigid and emotionally uninvested language when conveying bad news or conducting preoperative talks with a blank expression; prone to “information dumping” communication, the asymmetry in knowledge mastery between doctors and patients.	Physicians' use of computers in the examination room can affect patient-centered practice by diminishing dialogue (23). Physicians' use of jargon with diabetes patients with limited health literacy, creating barriers to understanding (24).
Cognitive biases	Habitual thinking patterns may contribute to cognitive biases such as anchoring effect (prematurely locking in an initial diagnosis) and availability bias (overestimating the probability of recently encountered diseases), thereby increasing the risk of diagnostic error (25).	Recent exposure to dengue fever increased the likelihood of misdiagnosing clinically similar cases as dengue fever, illustrating availability bias (26).
Stereotype-based clinical reasoning	Observable behavioral and decision-making patterns may arise when physicians rely on stereotypical assumptions rather than individualized patient assessment.	Perceiving the elderly as “poorly tolerant of treatment” and low-income individuals as “poorly compliant,” resulting in situations such as “cognitive avoidance,” “emotional isolation,” “excessive control,” and “defensive medicine” (27).
Avoidance of ethical discussion	When confronted with complex ethical dilemmas, physicians may defer to “regulations” or “legal mandates” to circumvent deeper, uncertainty-laden ethical discussion, resulting in moral apathy/oversimplification.	Physicians consistently prioritized conventional, law-based arguments, emphasizing legality and professional codes (28).

### 4.2 Dewey's view of moral development: dynamic experience and reflection

Dewey rejected morality as a static, pre-existing system of rules. Instead, he argued that morality is rooted in dynamic experience, which continuously evolves through reflection in response to conflicts, challenges, and choices that individuals encounter (21). He categorized three broad modes of human conduct: impulse/instinct–driven behavior, custom/convention–driven behavior, and reflective behavior. Impulse–driven behavior refers to actions arising from instincts and fundamental needs, which pertain to survival and reproduction. Custom–driven behavior occurs when individuals' conduct is regulated by social standards and behavioral norms. Such actions are less conscious, arising from uncritical, wholesale acceptance of group practices, and adherence to societal norms without questioning their validity. Reflective behavior emerges when individuals actively examine situations, evaluate potential consequences and value implications of their actions, and make deliberate choices grounded in reflection (29).

Dewey pointed out that moral growth is reflected in a movement from predominantly impulse- or custom-driven behavior towards more reflective behavior. That is, individuals become increasingly rational–capable of making value judgments and predicting consequences, increasingly socialized–able to coordinate diverse interests in social interactions, and increasingly moral–acting with concern for human flourishing and collective well-being (30). The core of this transformation is reflection, the disciplined examination of experience, the search for meaning, and the clarification of values.

### 4.3 Integrating reflective morality into competency-based medical education (CBME)

Patient-centered care requires physicians to move beyond established standardized protocols to understand and respond to patients' needs, values, and preferences. Emerging ethical challenges, such as those posed by AI-assisted diagnostics, cross-cultural communication, big data privacy, and uncertainty, require a capacity for critical reflection and ethical adaptability. Additionally, when facing high-stress environments and professional burnout, physicians must engage in self-reflection to rediscover meaning and value in their work, thereby contributing to the development of a resilient and ethically grounded professional identity. The development of these abilities is particularly relevant to CBME, especially to personalized competence, which includes personal skills, interests, habits, and convictions integrated with one's personality.

In Dewey's framework, reflective morality exhibits four essential characteristics: it positions individuals as autonomous moral agents; it demands contextual sensitivity to each situation; it requires critical examination of inherited customs and conventions; and it enables moral development through the iterative refinement of values based on experiential consequences. Grounded in this perspective, narrative medicine offers a principled pathway toward cultivating personalized competence. The connection between reflective morality and personalized competence is not simply that reflective individuals are necessarily more competent in every domain. Rather, reflective morality provides a developmental process through which physicians can examine how their existing habits, affective responses, values, and assumptions shape their professional judgments and actions. Deliberative capacities are continuously examined, refined, and integrated into purposeful action. Through this continuous reflection on experience, these personal dispositions can be reconsidered, refined, and reorganized in response to clinical situations. In this sense, reflective morality may contribute to the development of physicians' personalized competence by integrating personal dispositions with professional knowledge, contextual demands, and patient needs. The core practices of narrative medicine, close reading and reflective writing, provide structured opportunities for this process. By engaging physicians in situated and experiential reflection, these practices may visualize the implicit habits and responses, encourage reconsideration of established assumptions, and support the gradual development of more context-sensitive professional judgment. Reflective morality and personalized competence are therefore closely related but distinct developmental constructs. Reflective moral inquiry can shape how personalized competence is formed and expressed in practice.

## 5 Narrative medicine catalyzes physicians' personalized competence

Why should narrative practice be capable of activating the dynamic moral development that Dewey described? The answer lies in Dewey's theory of experience. In *Experience and Nature* (1925), Dewey argued that experience is an active, transactional engagement between the organism and its environment, carrying both cognitive and affective dimensions simultaneously. Through close reading or reflective writing, narratives constitute a situated, emotionally resonant, and meaning-making experience. When a physician reads a patient's illness narrative or writes about a morally challenging clinical encounter, they undergo experience in Dewey's sense, which may disrupt settled habits, mobilize affective impulses, and demand deliberative intelligence to restore coherence. Their moral significance emerges and awareness is aroused through interaction with existing habits and deliberative intelligence. This experiential structure, shared between Dewey's moral theory and narrative practices, provides the theoretical warrant for narrative medicine.

Narrative medicine provides a bridge for transitioning from customary morality to reflective morality. The core mechanism of narrative medicine lies in its systematic engagement with the physician's internal dynamic system of habit–impulse–intelligence. It creates a safe and reflective space in which physicians can critically examine their entrenched behaviors, corresponding to Dewey's habits; recognize and reflect on affective responses that may disrupt these habits, corresponding to impulses; and cultivate prudent moral judgment, which echoes intelligence amid value conflicts. These components are not independent or strictly sequential processes but interacting elements through which experience is examined, and professional responses may be reconstructed. This process of interaction, adjustment, and integration exemplifies the moral growth described in Dewey's ethical theory. Through repeated reflection on experience, physicians may move beyond awareness of their habitual assumptions and emotional responses toward reconsidering and modifying how they judge and act in particular clinical situations. Such reconstruction provides a potential link between reflective morality and the development of personalized competence. Narrative medicine thus offers a pathway for applying Dewey's philosophical insights to clinical practice, ultimately cultivating wiser, more compassionate, and more ethically accountable medical actions.

### 5.1 Reflecting on habitual behavior: narrative practice as a corrective to clinical habits

Through close reading of patients' illness narratives or writing parallel charts, obscure, messy, and tension-laden clinical experiences can be represented, so that they can be reconsidered and reanalyzed (31). Through such practices, physicians may realize how medical habits shape their responsed, thereby noticing aspects of clinical encounters they would overlook. Reading literary works such as *Better: A Surgeon's Notes on Performance* and *Being Mortal: Medicine and What Matters in the End* further enables physicians to examine the habitual patterns that pervade doctor–patient relationships, such as dependence on technology and emotional detachment, and the impact these patterns might have on patients' experiences.

Reflective writing encourages physicians to engage in independent thought, clarify their values, and assess potential consequences, and therefore serves as a form of rational and ethical training. It challenges preconceived beliefs and expectations (32), and demands that physicians take responsibility for their judgments and actions, rather than defaulting to regulations or authority. Clinician J. Trig Brown said that reflective writing enabled him to transcend evidence-based expertise by venturing into a realm of self-reflection, contemplation, and experiential mirroring (33). Narrative medicine examines internalized clinical habits, and uncovers the underlying irrationalities, biases, and contextual limitations. Through this reflective scrutiny, it may challenge igid patterns of customary morality, creating more nuanced and ethically sensitive perspectives.

### 5.2 Integrating affective impulse: developing moral responsiveness through affiliation

Narrative medicine has been shown to support the doctor-patient relationship by engaging with empathy, self-reflection, professionalism, and trust. Affective impulse does not equate with empathy or moral commitment itself. In Dewey's account, the significance of impulse emerges through interaction with existing habits and intelligence, rather than being inherently morally directed. Narrative practice emphasizes deep and generous listening. These encounters may evoke affective responses such as sympathy, discomfort, frustration, or emotional disturbance, which can interrupt habitual ways of perceiving a patient or clinical situation. Understanding patients' stories as unique individuals and reflecting on one's role and experience enables physicians to build a deeper sense of connection with their patients, which narrative medicine terms “affiliation.” This connection provides a relational and motivational basis for moral engagement when affective responses are interpreted and integrated through reflection and deliberation (34, 35). Through reflection and deliberation, physicians may examine their immediate responses together with the assumptions and habits that shape their interpretation. By integrating emotional energy and the motivation to care into the moral reasoning process, reflective morality emerges as a relational and compassionate form of ethics, more aligned with the nature of medicine.

### 5.3 Cultivating deliberative intelligence: contextual wisdom through reflective practice

Through close reading and writing, physicians are guided into contextualized reflection. The particular words patients use to describe their symptoms, their hesitation when selecting the treatment strategy, and the interaction among family members, all these details help the residents understand patients' lives, cultural background, and emotional triggers (31). Such practices train residents to attend to detail, interpret within context, and acknowledge individual differences. This may counter the mechanical application of rigid rules and enhance practitioners' capacity to determine what is good in each clinical situation (36). Moral growth is expected during the repeated contextualized reflection on such experiences. These methods will build narrative skills that support health care for patients, self-reflection and commitment to physicians' own health, care of colleagues, and more holistic approaches to medical practice, all of which together contribute to more sustainable and humane healthcare (20). By bridging the gap separating physicians from patients, themselves, colleagues, and society, narrative medicine facilitates physicians in offering accurate, engaged, authentic, and effective care for the sick. Deliberative intelligence, the capacity to reason wisely and responsively, is developed through sustained narrative practice.

### 5.4 Empirical evidence providing indirect but convergent support for the proposed framework

Studies of narrative medicine and reflective writing have reported improvements in reflective capacity, empathy, professionalism, and humanistic caring, providing empirical support for the proposed link between narrative engagement and reflective and affective processes (37–39). One pilot study used narrative medicine to engage medical students and overweight community members with the topic of weight bias and found that both groups reported benefiting from the experience through “depathologizing, humanizing, empathy-inducing, and power leveling interactions” (40). Prospective controlled trials among Iranian and Chinese interns and residents reported effects of narrative medicine courses on doctors' empathy (38, 41). The results showed that after the intervention, the empathy scores of the narrative medicine group were significantly higher than those of the traditional group. Meanwhile, majority of residents believed that the course effectively improved empathy and promoted self-reflection in medical practice. These findings provide preliminary empirical support for associations between narrative medicine training and empathy, reflective capacity, and communication-related outcomes. In addition, qualitative and quantitative studies of professional development have shown that reflective practice and narrative engagement are associated with professionalism learning and professional identity formation (42, 43).

Of particular relevance, Huang et al. identified self-exploration, reflection, and awareness of professional identity as mechanisms through which narrative medicine influenced professionalism learning, while empathy, communication, doctor–patient relationships, and understanding of patients emerged as outcomes (19). More recently, a large study of medical students found that reflective practice was significantly associated with professional identity formation, whereas self-reflection and insight alone were not (44). These findings suggest that reflection and enhanced deliberative intelligence may initially increase awareness; however, awareness cannot be the endpoint, instead, it is a starting point to potentially influence subsequent professional judgment and action. In a study of a narrative medicine curriculum including 130 medical students at Columbia University College of Physicians and Surgeons, a substantial number of students reported that the seminar enhanced their sense of well-being despite stress, burnout, and disillusionment with medicine (42). Although these studies do not directly validate the entire Deweyan moral growth pathway leading to personalized competence, these findings provide convergent support for individual links in the proposed framework.

### 5.5 Summary and a clinical example illustrating Deweyan moral reflection and the development of personalized competence through narrative medicine

Together, these three dynamically and iteratively interconnected mechanisms promote the progression from customary to reflective morality (Figure 1). Yet this moral transformation is not merely a philosophical achievement, it may also contribute to the development of personalized competence, which is individualized, experientially constructed, and not fully captured by standardized measurement alone. Personalized competence may be expressed in physicians' moral perception, empathic engagement, and the contextual wisdom they bring to ethically ambiguous encounters. Figure 2 is a clinical example illustrating the development of personalized competence through the dynamic Deweyan moral pathway. The growth of reflective morality and the continued development of personalized competence are not identical or automatically parallel processes. Reflective morality does not necessarily or directly improve all dimensions of personalized competence. However, it provides a developmental process through which these personal dispositions can be examined, refined, and progressively integrated with professional knowledge and contextual demands in clinical practice. Narrative medicine thus offers not only a framework of moral growth but also a practical pedagogy for cultivating the humanistically significant and the most difficult to teach, the personalized dimension of physician competence.

**Figure 1:**
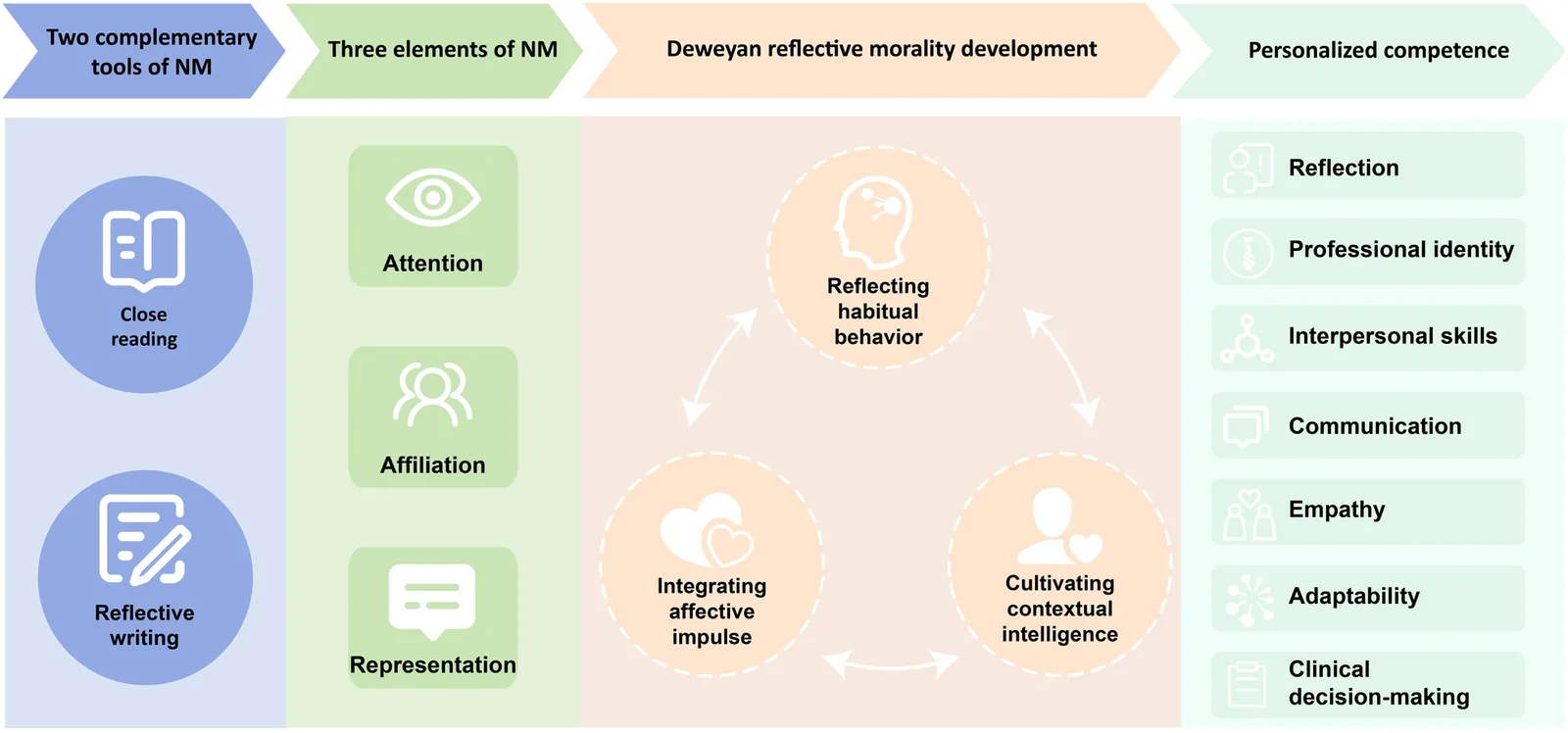
A Deweyan framework linking narrative medicine to the development of personalized competence.

**Figure 2:**
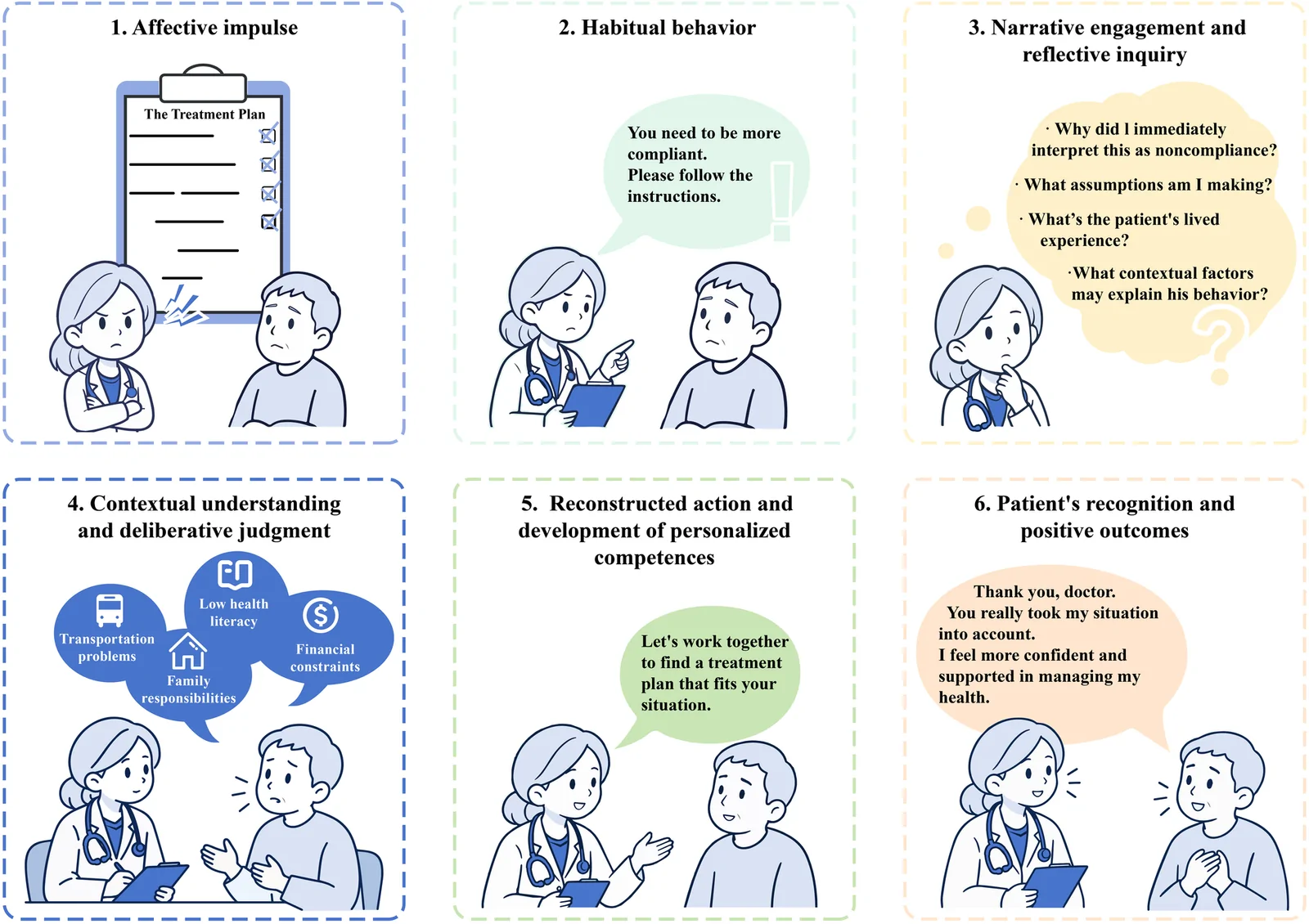
A clinical example illustrating the development of personalized competence through the Deweyan pathway from affective impulse to reconstructed professional action. **(1)** Affective impulse: An immediate affective response arises when the patient repeatedly does not follow the treatment plan. **(2)** Habitual behavior: Established habits may lead the physician to interpret the patient as “noncompliant” without further inquiry. **(3)** Narrative engagement and reflective inquiry: Narrative practices prompt examination of habitual interpretations and the patient's lived experience. **(4)** Contextual understanding and deliberative judgment: Deliberative inquiry broadens attention to contextual factors and enables reconsideration of the initial judgment. **(5)** Reconstructed action and personalized competence: Reflection and deliberation may contribute to more context-sensitive professional judgment and action. **(6)** Patient recognition and relational outcomes: The reconstructed response may strengthen trust and the physician–patient relationship.

## 6 Conclusion and limitations

This study conceptualizes narrative medicine through a Deweyan lens, and proposes reflective morality as a developmental framework for physicians' personalized competence. By engaging in the dynamic interplay of habit, impulse, and deliberative intelligence, narrative tools provide opportunities for learners to cultivate moral perception, empathic engagement, and context-sensitive judgment. This framework positions reflective morality not only as an ideological education, but also as a practical, teachable, and evaluable dimension of professional development. Future research should examine how narrative medicine interventions influence moral reasoning, decision-making, and patient-centered care practices among learners, and explore strategies for integrating these reflective practices into existing curricula. By bridging philosophical insight with educational practice, this approach offers a pathway toward more ethically attuned, compassionate, and competent physicians.

Limitations of this analysis should be acknowledged. The theoretical synthesis proposed here requires empirical validation through longitudinal studies examining whether narrative medicine training is associated with changes in physicians' moral reasoning from customary to reflective modes. Furthermore, implementation challenges, including curricular time constraints, institutional resistance, and the difficulty of assessing reflective moral development, warrant continued investigation. Several interventional studies cited in this paper are small pilot studies and should therefore be regarded as illustrative rather than confirmatory evidence for the proposed framework. We also acknowledge ongoing debates concerning the assessability of narrative and reflective capacities (6, 17), the cultural and interpretive assumptions underlying narrative practices (45), and whether narrative engagement necessarily promotes empathy and patient-centered care (46, 47). A comprehensive examination of these debates is beyond the scope of the present study.

### 6.1 Implications

Several implications follow from this analysis. For medical educators, this framework suggests that narrative medicine should not be treated as a supplementary humanities elective, but rather as a foundational component of CBME, particularly in cultivating the personalized dimension of professional competence that may be particularly important in actual clinical care. Contemporary CBME has achieved substantial progress in defining and assessing canonical competencies, yet the personalized dimension of competence, including moral perception, reflective judgment, empathy, and contextual sensitivity, remains difficult to capture through standardized measurement alone. This consideration may become increasingly relevant in the AI era. As AI becomes more integrated into clinical practice, medical education should continue to emphasize the humanistic and context-sensitive dimensions of competence, including moral perception, reflective judgment, empathy, and sensitivity to patients' individual values and lived experiences. Moreover, medical students' ethical development and its evolution as they enter clinical practice remain underexplored (48). Examining this development through the framework of personalized competence may help clarify how medical humanities and ethics-related courses can be effectively integrated into clinical practice.

For program designers, Dewey's tripartite model offers a theoretical scaffold for structuring narrative medicine curricula around experiences that challenge habitual assumptions, evoke affective responses, and prompt deliberative reflection. These dynamically interacting elements along with repeated narrative reflection support the development of context-sensitive ethical judgment. Reflective capacity is a core professional competency essential to patient-centered care. Integrating narrative practices into residency and undergraduate curricula may provide structured opportunities for learners to critically examine clinical habits, process emotionally challenging experiences, and develop context-sensitive ethical reasoning.

Dewey observed that “the good man is the man who, no matter how morally unworthy he has been, is moving to become better.” Medicine, at its core, is a moral enterprise sustained by commitment to such ongoing growth. By providing both the conceptual foundation and the practical tools for this developmental journey, the integration of Dewey's reflective morality with narrative medicine offers a compelling vision for physician formation, honoring the full complexity of what it means to be and to become, a moral practitioner.

## Data Availability

The original contributions presented in the study are included in the article/Supplementary Material, further inquiries can be directed to the corresponding authors.
